# Responses of Spring Barley to Zn- and Cd-Induced Stress: Morphometric Analysis and Cytotoxicity Assay

**DOI:** 10.3390/plants11233332

**Published:** 2022-12-01

**Authors:** Saglara Mandzhieva, Victor Chaplygin, Natalia Chernikova, Aleksey Fedorenko, Marina Voloshina, Tatiana Minkina, Vishnu D. Rajput, Maria Elinson, Ming Hung Wong

**Affiliations:** 1 Academy of Biology and Biotechnology, Southern Federal University, 344090 Rostov-on-Don, Russia; 2 Department of Biology, Bashkir State University, 450076 Ufa, Russia; 3 Consortium on Health, Environment, Education and Research (CHEER), The Education University of Hong Kong, 10 Lo Ping Road, Tai Po, Hong Kong, China

**Keywords:** heavy metals, *Hordeum vulgare*, polluted soil, toxicity, ultrastructural changes

## Abstract

Heavy metals such as cadmium (Cd) and zinc (Zn) could be dangerous and pollute the environment due to their high migration ability, robust bioavailability, and acute toxicity to soil biota and plants. Considering the above characteristics of these elements, the study’s aim was to explore the individual and combined impact of Cd and Zn contamination of Haplic Chernozem on growing two-row spring barley (*Hordeum vulgare* L.). The accumulation and distribution of Cd and Zn in various parts of *H. vulgare* have also been studied, which showed that Cd accumulation by *H. vulgare* occurred more intensely than that by Zn up to eight times. Cadmium and Zn suppress plant growth up to two times, more effect was noted by the combined impact of Cd and Zn. The study of plant morphological characteristics revealed that growth suppression and structural changes in the root and leaf tissues increased in proportion to Cd and Zn concentrations. Detailed analysis of the localizations of Zn and Cd in various organelles of *H. vulgare* cells was performed. Heavy metals change the ultrastructure of prominent energy-producing organelles in leaf cells, especially chloroplasts and mitochondria. Overall, the current findings offer insights into phytotoxicity induced by Cd and Zn individual application as well as in combination with the *H. vulgare* plant. Zinc showed protective effects against high doses of Cd under the combined application. These antagonistic interactions reduce their accessibility to *H. vulgare*. The present work can be useful in restricting the entry of these elements into the food chain and preventing creating a threat to human health.

## 1. Introduction

Due to the increase in population and anthrophonic activities, heavy metals (HMs) contamination in soil is increasing and affecting agricultural crop development via affecting the metabolic functions of the plants [1]. Cadmium (Cd) and zinc (Zn) are the most widespread and dangerous HMs pollutants in the environment due to their high migration ability, bioavailability, and toxicity [2]. In addition, Zn could be beneficial or toxic depending upon the concentration, dosage, and exposure time. The bioavailability of HMs in the soil is primarily governed by their binding to various minerals and organic matter compounds, precipitation, and chelation [3].

Cadmium primarily accumulates in the environment, is also a well-documented carcinogen, and is ranked seventh on the priority list created by the USEPA [4,5]. Due to extensive solubility, rapid mobility, lengthy half-life, and industrial and geological activity, Cd could primarily accumulate inside the food chain [6]. The presence of elevated concentrations of Cd, directly and indirectly, affects human health [7]. Cadmium is readily translocated to the consumable fractions of countless crops, thereby posing a substantial threat [4,8]. Plants are presumed to uptake Cd from the soils through their root systems, load it to the xylem, and then transport it to the aerial parts [9,10]. The molecular mechanisms of Cd accumulation in plants are uncertain [11]. It is therefore likely that several genes involved in these transport processes control Cd accumulation in plants [12]. In 2010, a single recessive gene located at qCdT7 that controls the rate of Cd translocation and accumulation in shoots of rice plants was identified [11]. Many works have been conducted to clarify the role of individual isogene on the transport of specific HMs in plants [13]. The competition between Cd and Zn/Cu was different to the competition between Cd and Fe/Mn in rice seedlings during metal transport within rice tissues, which is likely due to the difference in transporters and associated genes [14]. The HMs P1B-ATPases genes located in plasma membrane and tonoplast play more important role in metal translocation and storage within plant tissues than other proteins identified [14].

Zinc is considered a primary type of widespread soil pollutant in southern Russia [15]. An elevated dose of Zn in soils can enhance its uptake by plants, thereby affecting plant productivity [16,17]. The rise in mobile forms’ contents of Zn up to values exceeding the maximum permissible concentration (MPC) results in the downregulation of vital physiological processes, consequently affecting overall plant productivity [18,19]. Because chemically diverse pollutants coexist in the chemosphere, the transformation and accumulation of these pollutants by plants are altered. Various reports have previously demonstrated the antagonism between Cd and Zn uptake [20], as well as the synergism caused by the competition of the elements, mobilizing Cd and facilitating its transfer to the structure of the plant [21,22]. The toxicological characteristics of Cd are derived from its molecular resemblance to Zn, an essential micronutrient in biological networks derived for a myriad of biochemical and cellular functions [6]. For this reason, Zn theoretically decreases Cd-induced pressure by interacting with activating enzymes and growth hormones that control the growth and performance of the plant [23]. Numerous recent studies have demonstrated that Zn successfully reduces Cd uptake by plants because Cd and Zn have similar properties [24,25,26]. However, the physiochemical interactions of Zn and Cd, especially in biological systems, are still an issue of ongoing scientific discussion. Therefore, understanding the physiological and molecular mechanisms of Zn and Cd uptake, transport, and accumulation by plants is of great significance for formulating strategies for remediation of contaminated soils or crop preservation [27].

Plants can be a useful indicator of HMs contamination in an environment [28] in that root uptake of metals can integrate environmental levels across both spatial and temporal scales. In addition, HMs accumulation by vegetation can be further magnified within ecosystems via food webs. Roots not only prevent HMs from entering the plant but also enable their movement to above-ground plant tissues for accumulation into vacuoles, making them inert and hence non-reactive [29]. Identifying the resistance mechanisms to Zn and Cd in plants is essential for comprehensively identifying plant adaptation mechanisms against these metals.

The plant species of the *Poaceae* family accumulate Cd and Zn mainly in their roots [30], allowing them to survive longer in contaminated soils without being subjected to phytotoxic effects. In grains, phloem transport seems to be the principal mechanism that mediates Cd build-up throughout the reproductive phase [10,31]. This characteristic makes *Poaceae* family plants ideal for investigating the dynamics of HMs and their impact on plant morphology and ultrastructure at the expense of the gradual transfer of HMs from roots into vegetative and reproductive organs. Among the *Poaceae* family, spring barley (*Hordeum vulgare* L.) is one of the most critical food harvests widely used as food crops worldwide [32]. *H. vulgare* is recognized as an effective HMs accumulator with the capacity to accumulate Zn equivalent to a well-documented hyperaccumulator Indian mustard plant (*Brassica juncea*) [33]. It can be widely used as a bioindicator to assess the impact of maintenance and accumulation of potentially toxic elements [34]. The HMs build-up is prevented from accessing the plant’s aerial parts. It does not interfere with or disrupt the basic processes of the plant, such as growth and development [35].

The resistant potential of *H. vulgare* for the two most prominent HMs, Zn and Cd, should be investigated rigorously. The aim of this work was to study the effect of diverse doses of Cd and Zn (individual and combined) on the *H. vulgare* (grown in Haplic Chernozem) by observing parameters of plant growth, quantitative analysis of Zn and Cd in soil, metal accumulation in plant tissues, morphological and ultracellular alterations.

## 2. Results

### 2.1. Total Content and Exchangeable Forms of Metals in the Soil

The results of measurements of total and exchangeable forms of metals in the soil are given in Table 1. As shown, the total content of Zn and Cd in the control samples without any treatment was 68 mg/kg and 0.27 mg/kg, respectively. The exchangeable form of Zn and Cd concentrations were 1.1% and 7.4% of the total content in the control, respectively. When 440 mg/kg and 1100 mg/kg of Zn were added, the total content increased by 8 and 17 times, respectively, as compared with the control. The concentration of the exchangeable form significantly increased 39-fold and 108-fold, respectively, with respect to the control (Table 1). The concentration of Zn exchangeable form was 5.3% of the total content (when adding 440 mg/kg) and 6.5% (when adding 1100 mg/kg). The total Cd content increased by 15 and 37 times than in the control samples. When 4 mg/kg and 10 mg/kg of Cd were added to the soil, the exchangeable form concentration increased 43 and 114 times, respectively (Table 1). The Cd exchangeable form was 20.3% of the total content (when adding 4 mg/kg) and 22.6% of the total content (when adding 10 mg/kg).

With the combined addition of Zn and Cd at different concentrations (2 and 5 MPC) in the soil, the exchangeable metal concentrations increased compared to the individual metal contamination. In the treatment with 2 and 5 MPC of combined metals, Zn and Cd exchangeable form concentrations were 8% and 11.8% of total content, and 27.2% and 34.8% of total content, respectively. For both types of contamination, Zn was characterized by significantly lower mobility than Cd (Table 1). Under the conditions of combined metal contamination, Zn fixation ion exchange processes were more involved. This indicates the weak ability of Zn to compete for the adsorption spaces, as its mobility efficiently improves in the presence of Cd.

### 2.2. Zn and Cd Accumulation and Distribution in H. vulgare Tissues

**Figure 1:**
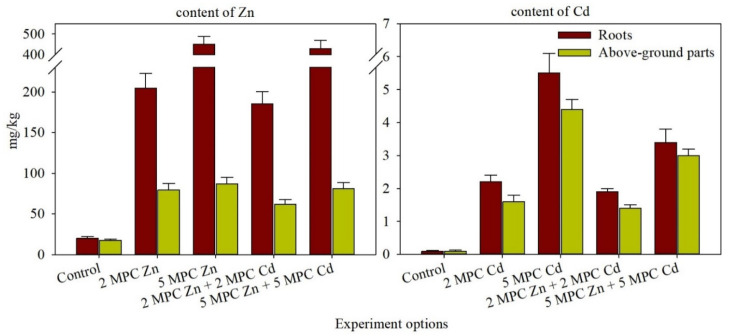
The metal content in various parts of *H. vulgare* grown in the soil spiked with different levels of Zn and Cd, individual and with combined metals, mg/kg. Above-ground parts of plants include stems and leaves.

Adding Zn salts to the soil of the model experiment led to metal accumulation in the above-ground parts of the plants, increasing from 79 mg/kg (1.2 MPC) to 87 mg/kg (1.7 MPC) (Figure 1). The highest Zn concentration was observed in with 1100 mg/kg treatment group. Consequently, up to five times increased in the Zn content of the above-ground parts and 22.6 times in the plant roots compared to the control. Higher Cd mobility in the soil compared to Zn resulted in its significant accumulation in the plants. Thus, Cd concentration under the conditions of the higher soil contamination level (5 MPC) significantly increased in comparison to the control by as much as 44-fold in the above-ground parts (14.7 MPC) and 39-fold in the roots (Figure 1). HMs accumulation was mainly observed in the plant root system in all treatments. The contents of Zn and Cd in the roots and above-ground tissues varied according to their concentrations in the soil.

### 2.3. Morphological and Ultrastructural Changes in the H. vulgare

**Figure 2:**
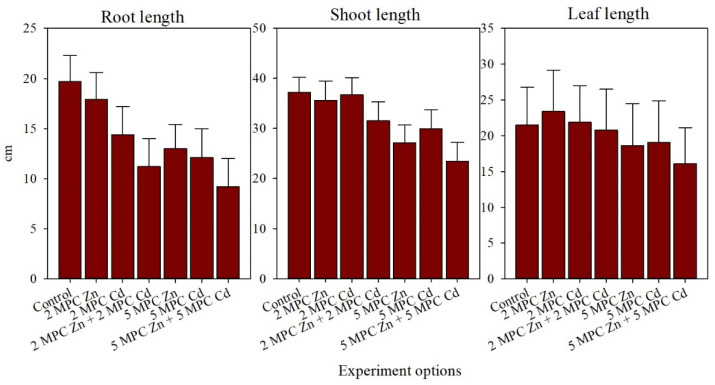
Effects on morphological characteristics of *H. vulgare* in different treatments of the model experiment, cm.

The height of control plants during the stem elongation phase was 37.2 cm, and the lengths of the roots and leaves were 19.7 and 21.5 cm, respectively (Figure 2). No noticeable effect of Zn and Cd (2 MPC, together and separately) on the morphological characteristics of the plants was observed. However, with an increase in soil HMs concentration up to 5 MPC, plant growth suppression was observed. The greatest inhibitory effects were observed in the combined metal contamination, where the root length and plant height were reduced by 53% and 37%, respectively.

**Figure 3:**
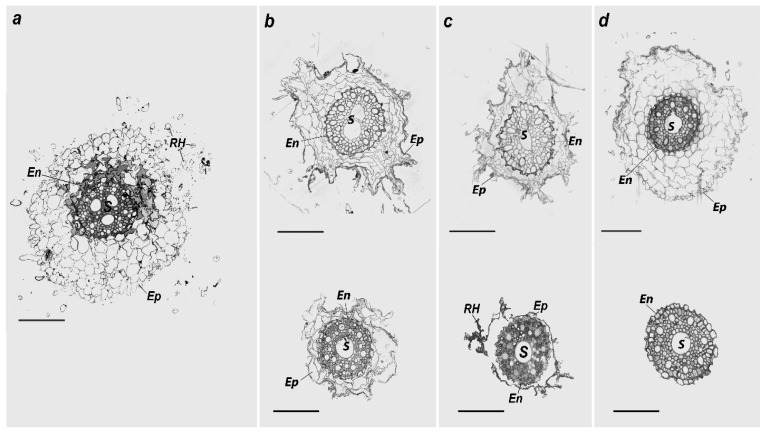
The *H. vulgare* root cross-section: (**a**)—control, (**b**)—treatment with Zn 440 mg/kg (top), and Zn 1100 mg/kg (bottom) (**с**)—treatment with Cd 4 mg/kg (top) and Cd 10 mg/kg (bottom), (**d**)—treatment with combined contamination by Zn 440 mg/kg + Cd 4 mg/kg (top) and Zn 1100 mg/kg + Cd 10 mg/kg (bottom). RH—root hairs, Ep—epidermis, En—endodermis, S—central cylinder. Scale of the section was 100 µm.

Microscopic observations showed that the exterior surface of the control roots was covered with a monolayer of the epidermis with root hairs 150–200 µm2 long (Figure 3a). Under the epidermis layer, there were several layers of parenchymal cells with a size of 445 μm^2^, most of which were corked. The central part of the root cross-section was occupied by the central cylinder separated from the epidermis by the endodermis, containing a layer of thick cells, thickening radial, and cross-sectional walls. The central cylinder included pericycle and conductive tissues and occupied 13,800 μm^2^ on average. Pericycle cells protruded slightly from the radius and had a thin cell envelope. The large vessels of the xylem were surrounded by living cells of the xylem parenchyma. Large pores in the cell walls connect them with the vessels. The phloem contained sieve-type pipes, satellite cells, and parenchyma. While the root cross-sectional area was 84,472 μm^2^, the main part was occupied by the epidermis (Table 2).

In the experimental treatments with individual metal contamination with Zn, the root structure of *H. vulgare* had distinctive qualitative anatomical differences from the control samples. The number and length of root hairs primarily decreased (Figure 3b). The parenchyma layer cells were deformed and disorganized compared with the control cells. In general, there were no cytoplasm or organelles, and they contained only cell walls. The cell walls’ integrity in some sections was disturbed, and some cells were united into one cavity. An inversely proportional relationship was observed between the cross-sectional area of the epidermis and the average size of the cells and the pollutant concentration in the soil (Table 2). The central cylinder occupied a relatively more prominent part of the total root cross-section than the control. In contrast, the radial-type built vessel bundles with unclearly exposed radial symmetry. Additionally, the central cylinder had signs of a secondary structure with several vessels in the center at a Zn contamination level of 2 MPC. It should be noted that large vessels in the central part were observed, from which minor phloem and xylem bundles protruded in the Zn treatment at 5 MPC. In addition, the xylem ray vessels had a larger clearance toward the periphery.

With only individual metal contamination with Cd, the anatomical root structure differed entirely from the control samples and the plants grown with Zn contamination. The epidermis cells were strongly differentiated, and most did not have cytoplasm, only cell walls (Figure 3c). The epidermis had rarer and shorter root hairs than that of the control. The parenchyma cells were powerfully decreased and contained a single layer of cells (in some sections, none were present). The cross-sectional area of the epidermis decreased by increasing Zn and Cd contents in the soil (Table 2). The average size of parenchyma cells with the Cd contamination level of 2 MPC was found to be smaller than both samples, including the control and the treatment with 2 MPC Zn. At the contamination level of 5 MPC Cd, the size of the epidermis cells could not be measured owing to heavy reduction. Disruption of the endodermis layer structure was not observed. The central cylinder occupied a more significant part of the root’s total cross-section than the control and treatments with individual metal Zn contamination in the soil. In addition, the total area of the conducting tissue was the same as that of the control. The vascular bundles were of the radial form. The smaller phloem and xylem bundles went radially from one substantial vascular bundle in the cylinder’s central shaft.

The anatomical structure of the *H. vulgare* roots grown under combined Cd and Zn contamination differed from that of the control samples and the plants grown with individual metal contamination. In the treatment with 440 mg/kg of Zn + 4 mg/kg of Cd, the outer epidermis layer in the roots of plants had living cells with few root hairs (Figure 3d). Root hairs were observed less frequently than those in the control and other treatments with individual metal contamination. However, the epidermis cells had a structure and volume similar to those of the control. However, they were clearly more established than in the Zn and Cd-contaminated individual samples (Table 2). On average, the cells in this layer were larger than those in other contaminated samples, and many cells had intact cytoplasm due to more secondary cell wall damage. In addition, endodermal cells had different sizes and shapes, and some endodermal cells were intact. The central cylinder area was smaller than that of the control and all the other treatments. As in the other samples, the radial-type built vascular bundles, but the radial symmetry was not clearly expressed. With contamination at the Zn 5 MPC + Cd 5 MPC rate, the epidermis layer with the root hairs and the epidermis were fully degraded. The structure of the endodermal layer was also damaged. In the inner circumference of the central cylinder, there was one sizable vascular bundle, around which vascular bundles with clearly expressed radial symmetry were located.

**Figure 4:**
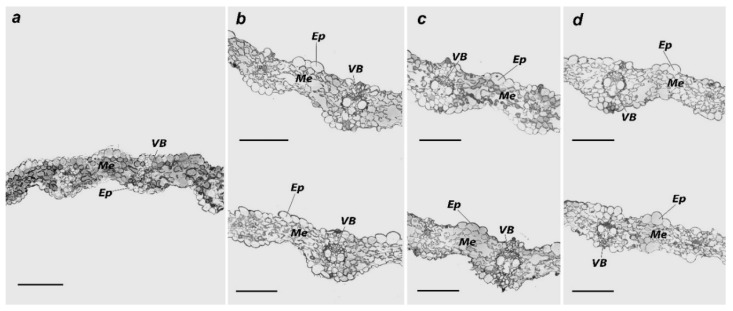
The cross-section of the *H. vulgare* leaf lamina. (**a**)—control, (**b**)—treatment with 440 mg/kg of Zn (top) and 1100 mg/kg of Zn (bottom), (**c**)—treatment with 4 mg/kg of Cd (top) and 10 mg/kg of Cd (bottom), (**d**)—treatment with combined contamination by 440 mg/kg of Zn + 4 mg/kg of Cd (top) and 1100 mg/kg of Zn + 10 mg/kg of Cd (bottom). Ep—epidermis, Me—mesophyll, VB—vascular bundle. The scale of all the sections was 100 µm.

The structure of the leaf lamina in control was characterized by an orderly organization and even cell localization in the leaf chlorenchyma (Figure 4a). The average area of the chlorenchyma cells was 124 µm^2^ with 6–7 laminas (Table 3). Additionally, some evidence of the division of mesophyll cells into palisade and spongy parenchyma could be seen. The average number of units in 1 μm^2^ was 2981. The palisade parenchyma cells were located in a single row of the upper epidermal layer. They occupied approximately a quarter of the leaf lamina cross-section area. In addition, they had rounded shapes and were tightly attached to one another. Spongy parenchyma cells were located in the central and lower parts of the leaf and adhered to the lower epidermis. Cell shape was observed to be more protruded. The hallmark of their spatial organizations was a minor part of the tightly contacting cells and availability of broad intercellular spaces.

**Figure 5:**
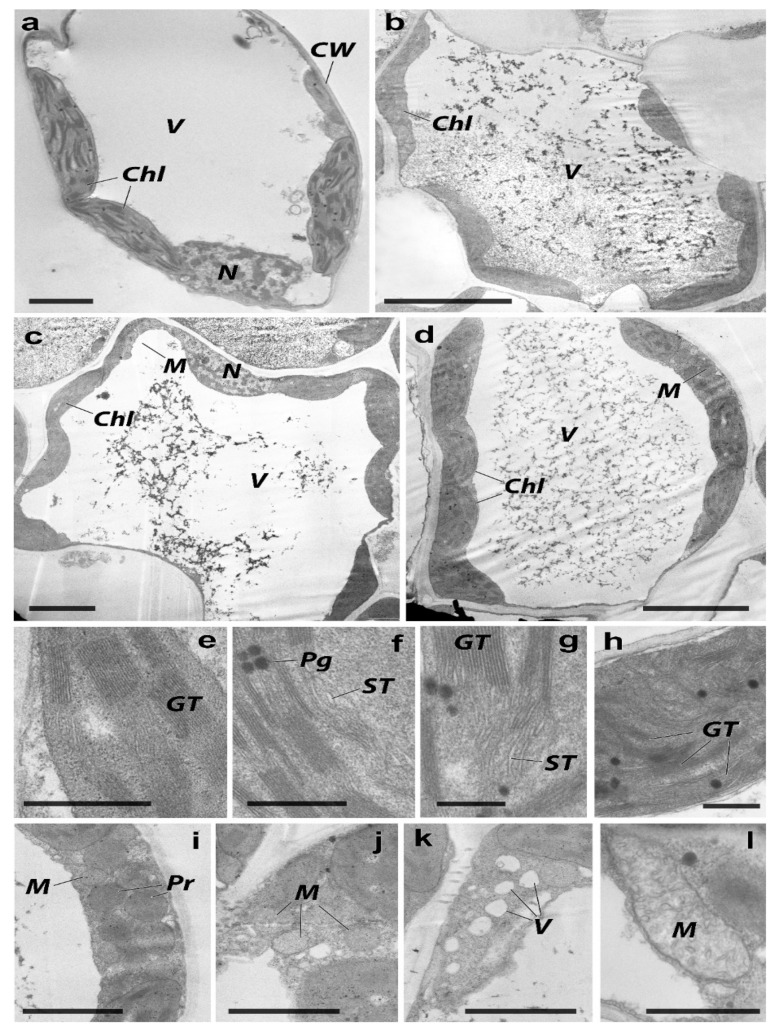
TEM images of *H. vulgare* leaf chlorenchyma. (**a**,**e**)—control, (**b**,**f**,**j**)—treatment with 1100 mg/kg of Zn, (**c**,**g**,**k**)—treatment with 10 mg/kg of Cd, (**d**,**h**,**i**,**l**)—treatment with combined contamination of 1100 mg/kg of Zn + 10 mg/kg of Cd. M—mitochondria, CW—cell wall, Chl—chloroplast, Pg—plastoglobuli, GT—granal thylakoids, ST—stroma thylakoids, N—nucleus, M—mitochondria, Pr—peroxisome, V—vacuole. Scale segment (μm): a-2, b-5, c-5, d-5, e-0.5, f-0.5, g-0.3, h-0.5, i-2, j-2, k-2, l-1.

Individual and combined metal soil contamination led to slight alterations in the ultrastructure and morphology of the leaf lamina compared to plants grown on uncontaminated soil. Table 3 displays a rise in the intercellular space and a reduction in the orderliness of the spatial organizations of parenchymal cells. In the control, the parenchyma, chlorenchyma cells, and conductive leaf bundles of the xylem had normal ultrastructural characteristics (Figure 5a). Prominent energy-producing organelles were found in triads in the lumen of the cytoplasm. With a diameter of 0.1 to 0.2 μm, chloroplasts were lenticular primarily in form, abundant in grains, and few in plastoglobuli. Over the whole region of the plastid cut, grains containing up to 25–30 units of thylakoids per grain were spread equally (Figure 5e). An electron-light matrix and teardrop-shaped cristae were present in the rounded mitochondria. Round peroxisomes (0.3–0.8 μm) had a well-defined single membrane. Granular matrix and filamentous inclusions were present inside the peroxisomes. The nucleus was oval and elongated, whereas the nucleolus was not expressed. Small chromatin blocks were evenly distributed throughout the karyoplasm.

Ultrastructural changes caused by Zn were mainly observed in energy-generating organelles in leaf cells (Figure 5b). In chloroplasts, there was a slight increase in electron-dense matrices. The organelles became less elongated and predominantly had rounded shapes. The membrane apparatus was unevenly distributed in the stroma of the plastid and less ordered. Thylakoids in the stroma were swollen (Figure 5f,g), and the structure of the mitochondria was visibly altered in comparison to that of the control. In addition, peroxisomes were enlarged and lodged with electron-dense content. The tendency to spatially rearrange energy-producing organelles increased pollution levels. In the control, the plastids, mitochondria, and peroxisomes were evenly distributed and were found in triads in the cytoplasm. In contrast, upon exposure to Zn, the mitochondria and peroxisomes were collected in 5–10 units.

The cytoplasm was saturated with ribosomes, and the endoplasmic reticulum was visibly swollen (Figure 5j). Similar ultrastructural changes in the cellular apparatus were triggered by Cd exposure, as Zn case (Figure 5c). The chloroplasts were more electrically charged and rounded than those in the control group. The membrane apparatus of the plastids was less organized, and the thylakoids were moderately swollen. The mitochondria and peroxisomes did not exhibit visible structural modifications, but their spatial rearrangement was observed. In addition, the endoplasmic reticulum was swollen, and the cytoplasm was vacuolized (Figure 5k). Substantial changes in the ultrastructure of *H. vulgare* leaf cells were observed during the combined Zn and Cd contamination (Figure 5d). The number of chloroplasts was lower than that of the control. Their shape was oval, and they had an electrically dense matrix. Numerous plastoglobuli were grouped into 3–4 units in the plastid stroma. More importantly, the membrane apparatus was disorganized. The granules were small and unevenly distributed throughout the organelle area. A single swollen mitochondrion containing randomly oriented crystals with a highly charged matrix was observed (Figure 5l). Peroxisomes were larger than mitochondria and had a dense matrix. The grouping tendency of mitochondria and peroxisomes by 8–10 units was observed between plastids (Figure 5i).

Thus, heavy metals changed the ultrastructure of energy-producing organelles in leaf cells, especially chloroplasts and mitochondria.

## 3. Discussion

The simultaneous presence of some metals in the ion system leads to competition among HMs for their ability to interact with definite types of reaction centers [36]. In the present experiment, Zn showed a stronger fixation strength and a lower degree of mobility than Cd in the soil. Cations of Zn and Cd influence their sorption at the mutual presence in the soil solution and are directly exposed through competition for adsorption sites. In competitive conditions, HMs are less strongly sorbed by soil-limited sorption sites [37].

With increasing soil contamination by HMs, the level of mobile forms of HMs also became more significant, which shows a decrease in the protective functions of soils against pollutants [38,39]. The combined treatment with Zn and Cd had a lower plant metal concentration than the individual treatments shown in this work, as well as in earlier studies [40,41,42]. This is probably related to the antagonism between Zn and Cd. Therefore, the treatment had a lower plant metal concentration. The co-exposure of Zn and Cd to *Salvia sclarea* plants increased Fe and Mg content in the leaf with respect to control, whereas plants treated with Cd only showed high levels of Ca, Mn, and Cu [43]. These changes in metal accumulation are likely to be because of the defense mechanisms of excess Zn against Cd toxicity [44]. Zinc accumulation in meristic cells of the root has also been observed in other cereals, including wheat [45,46]. It is noted that Zn acts as a functional, structural, or regulatory cofactor of many photosynthetic enzymes [47]. In the present work, 1.4–1.7 times higher Zn was accumulated than in the lower-raw leaves. It showed that the microelements were concentrated in places where they were vital for plant organ functioning. Under Zn-deficient conditions, Zn accumulation in roots was almost half that in stems and leaves and only approximately 25% in grains of wheat [48].

The intensity of Cd accumulation by *H. vulgare* in the model experiment was higher than that of Zn accumulation. However, soil contamination by Cd in the range of 0.5–100 mg/kg did not cause any visible changes in the condition of plants, although it led to some significant aberrations in the number of morphophysiological indices. The toxic effect produced upon morphometry and the ultrastructure of the plant tissue depended on the concentration of Cd in the soil [49]. The increase in Cd concentration in the soil led to a severe reduction in plant root length compared to Zn. However, a Cd content of 10 mg/kg did not affect the plant height or the fresh and dry weights of the *H. vulgare* plants. An increase in the Cd concentration in sod-podzolic soil severely affected the morphometric characteristics of the barley [50].

Minor differences in *H. vulgare* morphometric indices among the treatments were also observed [50]. However, Cd at 10 mg/kg considerably affected all the morphological indices of the plants, lowering them by 55–75%. Our results also indicated that morphological root parameters were considerably modulated under high Cd stress. Inhibition of root extension, lateral root development, and induction of root tip radial swelling were the main root morphological changes induced by Cd. Earlier toxic effects of Cd on the root morphology of durum wheat were found as increases in the number of tips and primary root length, damages in the cell membrane system, and thickness in the cell wall [51]. According to recent findings, cell walls are essential for improving plant Cd tolerance because they restrict the metal from entering the root cells [52,53]. Further, our work indicates that the internal root structures were also damaged by Cd stress. The vascular tissues’ elevated lignification, cell cycle slowdown, particularly in meristematic cells, and disorganized cell configurations in the lateral roots were a few other examples [54,55]. Plant responses to lower Cd toxicity might include thicker root tips and radially enlarged cells [56]. The maximum damage to the exodermis cells was observed in the high Cd contamination individually and in combination with Zn. In a similar study on radish (*Raphanus sativus* L.), the joint antagonistic activity of Cd and Zn was demonstrated on exodermis cells [57]. The root epidermis cells are the major sites for the absorption of HMs, which slows their transfer to other plant parts. Studies on stonecrop (*Sedum alfredii* Hanse) and maize (*Zea mays* L.) pointed toward similar mechanisms for reducing free Cd^2+^ by accumulating it in the cell walls and intercellular spaces [58,59]. There were only some weak quantitative morphometric differences compared with the control, which were expressed by increasing the parenchyma intercellular space and disorganizeds arrangement of parenchyma cells.

Due to co-contamination, the accumulation of Cd in plants’ edible sections might be greatly reduced by a very high and nearby phytotoxic level of Zn content [60,61,62]. Because of rivalry in the soil, at the surface of the roots, and/or within the plant system, Zn has both antagonistic and synergistic effects on the absorption of Cd by plants [24,40,41]. This may prevent Cd uptake, which reduces Cd accumulation. In particular, a comparable transport mechanism on the root plasma membrane mediates the absorption of Cd and Zn in soft and durum wheat seedlings [63]. Through a variety of ways, these researchers concluded that Zn might dramatically boost growth and decrease Cd accumulation: reducing oxidative stress [42], enhancing photosynthetic performance [64], and stimulating nutrient balance [25]. Changes in intercellular levels were also observed in the sequential reduction of the lamina number per cell. The structural disorganization of the chloroplast apparatus in sub-cellular tissues of *H. vulgare* induced by Cd and Zn, a rise in the plastoglobuli amount, and other intercellular changes in the leaf chlorenchyma due to Zn-induced oxidative stress were found. Damages in nuclei, mitochondria, and chloroplasts of *Beta vulgaris* were induced by Cd stress [65]. However, excess Zn^2+^ input reduced Cd^2+^ toxicity in *Salvia sclarea* L. and helped to restore chloroplast ultrastructural organization [44]. The accessibility of metals under combined contamination to the plants was lower than that under individual metal contamination. Despite the high competition and antagonism of metals for uptake/aggregation in the plant system, the synergistic effects would impose more aggravated risks than individual metals.

## 4. Materials and Methods

### 4.1. Model Experiment

For the model experiment, Haplic Chernozem was used as a control. The topsoil (0–20 cm) was taken from the protected area of the Persianovskaya Zapovednaya Steppe in southern Russia. Soil sampling was performed according to ISO 10381-1 (2002) [66]. The soil was characterized by the following indicators: the clay content (soil particles ˂ 0.001 mm), 52%; silt (soil particles > 0.001 mm), 30%; content of C_org_, 2.4%; CaCO_3_, 0.4%; cation exchange capacity (CEC), 33 cmol (+)/kg; and pH, 7.5. Soil particle size distribution was determined according to ISO 13317-2 (2001). The pH of the 1:5 soil:water suspension was measured using a glass electrode according to ISO 10390 (2005). The carbonate content was determined by the volumetric method using a Scheibler apparatus, according to ISO 10693 (1995). The total organic carbon (Corg) content was determined using sulfochromic oxidation, ISO 14235 (1998). The cation exchange capacity (CEC) was analyzed using 1 M NH_4_OAc after soil saturation with 1 M BaCl_2_ (pH 6.5) (ISO 11260:2018).

The selected soil was manually cleaned from the plant leftovers, then filtered by a diameter of two mm opening sieve. The vegetation pots 3 L sized with a locked draining arrangement were packed with 2 kg of soil artificially contaminated with Cd and Zn. The amounts of introduced pollutants were 4 mg/kg (2 MPC) and 10 mg/kg (5 MPC) Cd, and 440 mg/kg (2 MPC) and 1100 mg/kg (5 MPC) Zn (SanPiN 1.2.3685-21). These doses of Zn and Cd were considered due to the existing doses of these metals in soils adjacent to industrial enterprises of the Rostov region as well as other parts of Russian Federation soils [67,68,69]. The individual and combined introduction of Cd and Zn into the soil was performed in the form of CdSO_4_ and ZnSO_4_. These salts are the main forms in which metals enter the soil from technogenic sources. Dry metal salts were added and thoroughly mixed with soil. The incubation period of contaminated soil was one month while maintaining the humidity with the regular application of distilled water in the pots at 60% of the total field moisture capacity. Contaminated soil was exposed at room temperature and natural lighting. The whole experiment was performed in triplicates.

The experimental scheme was as follows:

(1) Control;
(2) 2 MPC: 440 mg/kg Zn;
(3) 2 MPC: 4 mg/kg Cd;
(4) 2 MPC: 440 mg/kg Zn + 4 mg/kg Cd;
(5) 5 MPC: 1100 mg/kg Zn;
(6) 5 MPC: 10 mg/kg Cd;
(7) 5 MPC: 1100 mg/kg Zn + 10 mg/kg Cd.

After the incubation period of one month, 20 grains of *Hordeum vulgare* L. were sown per pot. *Hordeum vulgare* L. distichum cv. Ratnic, a widely cultivated species in the Rostov region (Russia), was used as a test culture. *H. vulgare* is used for bio-testing [66]. This variety was created in 2004 intraspecific hybridization method. Zoned in the South of Russia due to high drought tolerance and disease resistance. Vegetative growth of plants occurred under photo lamp lighting (14/10 h light–dark cycle) at 25 ± 3 °C. The lowest field moisture capacity was maintained in the soil for vegetative development. No additional fertilizers or nutrients were applied. Plants were selected for further detailed experiments 37 days after planting in the stem elongation phase when *H. vulgare* absorbed most of the elements from the soil during the entire growing season and had a well-developed green vegetative biomass. Twenty plants were sampled from vegetation pots.

### 4.2. Quantitative Analysis of Zn and Cd in Soil

The total content of HMs, which includes all metal compounds, in the soils at different pollution levels was determined by X-ray fluorescence (Spectroscan MAKC-GV spectrometer NPO Spectron, Russia). To extract exchangeable parts of HMs from soil samples, which characterize the bioavailable compounds of metals, 1 N ammonium acetate CH_3_COONH_4_ buffer was used at a pH of 4.8 [70]. The filtrates were measured using an atomic absorption spectrophotometer (AAS) with flame atomizations (“Kvant-2” Kortek, Russia).

### 4.3. Quantitative Analysis of Zn and Cd in Plants

Before being divided into sections, fresh plants were thoroughly cleansed of dirt and plant debris using flowing distilled water. The collected samples were homogenized, dried at room temperature, then cut into short, homogeneous pieces using a plastic knife. The air-dried 1 g samples of *H. vulgare* tissues were dried up in an oven at 65 ± 5 °C. The dried samples were then ashed using a muffle furnace at 450 °C for six hours [71]. The ash was dissolved in 5 mL of 20% HCl and filtered through 0.45 mL of Whatman filter paper. The final volume of the extracts was made using deionized water up to 50 mL before the AAS measurements. All analyses were performed in triplicates, and the mean results were stated. Because the HM concentration in plants is usually at a trace level, the use of AAS provides reliable outcomes because of its ability to reconcile high sensitivity and low limits of detection [71].

Soil and plant contamination was assessed by comparing the MPC of these elements according to the Russian hygienic standards (SanPiN 1.2.3685-21).

### 4.4. Morphometric, Microscopy, and Cytomorphometric Analysis

The growth and development of *H. vulgare* and changes in plant tissues and cells were studied. The plant height, root length, and shoot length were measured. For microscopic examination of the tissues and cells of *H. vulgare*, cuttings from the middle part of the second or third leaf (2 × 2 mm) and 2 mm of root (middle part) samples were collected, especially from the root hair zone. The fixation of samples was performed using a 2.5% solution of glutaraldehyde in phosphate buffer (pH 7.4) for two h under vacuum pressure at room temperature. The samples were washed twice with sucrose phosphate buffer, fixed in 1% OsO4 solution, and incubated for 2 h. Then, the plant samples were dehydrated with increasing ethanol concentrations (50%, 70%, and 96%). Before transferring to 96% alcohol, the samples were placed in a 70% alcohol solution of uranyl acetate for 12 h at +4 °C. This was followed by washing with uranyl acetate and continued dehydration in absolute ethanol and acetone for three shifts of 15 min each. The leaf cuttings were impregnated with resin in a series of Epon solutions in acetone at increasing concentrations and poured into Epon. A thermostat was used for polymerization at 37 °C (1 d) and 60 °C (2 days). All stages of tissue preparation for morphological observation (contrast, dehydration, imprisonment in Epon-type polymerizing mixtures, preparation, and staining of semi-thin sections for light-optical study) were performed using standard methodological techniques (Fedorenko et al., 2018). Semi-thin sections of 0.5–1 μm for light-optical observation were stained with methylene blue. They were then placed under a light-optical microscope (LOMO, St. Petersburg, Russia) at magnifications of ×100 and ×400 for observations. Ultrathin sections were prepared by microtome (Leica EM UC6, Wetzlar, Germany) and examined by light-optical microscopy (Mikmed-6, St. Petersburg, Russia) and TEM (Tecnai G2 Spirit Bio Twin, Amsterdam, The Netherlands).

Cytomorphometric analysis was performed by a light-optical microscope for *H. vulgare* roots. This included measuring the root and central cylinder cross-section area and calculating the cross-section root area. Optical images of the leaf plates were also analyzed. These included counting the number of mesenchymal cells per mm^2^, the ratio of the cell area to the intercellular space, measuring the average size of the mesenchymal cells, and the number of plastids in the parenchyma cells. An Olympus Soft Imaging Solutions ITEM was used to measure cell fragments.

### 4.5. Statistical Analysis

All data were processed using STATISTICA 10.0 software. The Shapiro–Wilk test was used to determine if the data were normal. The parametric test ANOVA was used. The mean, median, minimum and maximum values, standard deviation (SD), and coefficients of variation (CV) were computed as descriptive statistics. Values from several analyses are shown as mean SD.

## 5. Conclusions

The negative effects of Cd and Zn contamination were noted in *H. vulgare* grown in Haplic Chernozem. The outcome of exposure to Zn and Cd on the tissue morphometry and ultrastructure of plants first depends on the metal, second on the level of its concentration, and finally on the type of soil contamination. The maximum damage to plant tissues was observed with the highest Cd and Zn combined contamination in the soil. However, higher Cd soil content led to a more significant decrease in plant root length than Zn. Moreover, it was noted that the synergistic consequence of the Cd and Zn elevated threat compared to individual metal contamination. These findings deepen our understanding of heavy metals behavior in agricultural soils to develop an efficient mechanism that restricts their mobility and accessibility to plant tissues. Zinc and Cd accumulation in the soil system, as well as in edible crops, is a concerning issue and constantly increasing due to anthropogenic activities. Therefore, further advancement is required to formulate effective strategies to mitigate Cd and Zn toxicity, individually and in combination.

## Figures and Tables

**Table 1:** Zinc and Cd total content and exchangeable form concentrations in soil exposed to spiking individual or combined metals, mg/kg.

Rate of Application	Zn		Cd	
	Total Content	Exchangeable Form Concentration	Total Content	Exchangeable Form Concentration
Control	68 ± 7	0.7 ± 0.1	0.27 ± 0.02	0.02 ± 0.002
2 MPC	512 ± 62 *	27.1 ± 2 *	4.18 ± 0.31 *	0.85 ± 0.11 *
5 MPC	1154 ± 104 *	75.4 ± 6 *	10.05 ± 0.93 *	2.27 ± 0.22 *
2 MPC Zn + 2 MPC Cd	487 ± 35 *	39.0 ± 3 *	4.23 ± 0.31 *	1.15 ± 0.10 *
5 MPC Zn + 5 MPC Сd	1142 ± 99 *	135.0 ± 9 *	10.11 ± 0.92 *	3.52 ± 0.30 *

± standard deviation (SD); * significant at the 0.050 probability level.

**Table 2:** Cytomorphometric indices of the *H. vulgare* grown at different levels and contamination of the soil by Zn and Cd, µm^2.^

Rate of HM Application, mg/кg	Root Cross-section Area	Central Cylinder Cross-Section Area	Ratio Between Root Cross-Section and Total Area	Ratio of Epidermis to Total Area	Medium Size of the Epidermis Cell
Control	84472 ± 2254	13800 ± 527	6.1	5.1	445 ± 37
2 MPC Zn	49211 ± 1931 *	15535 ± 642 *	3.2	2.2	337 ± 39 *
5 MPC Zn	29143 ± 958 *	10744 ± 527 *	2.7	1.7	207 ± 46 *
2 MPC Cd	28832 ± 821 *	12361 ± 610 *	2.3	1.3	213 ± 59 *
5 MPC Cd	22161 ± 798 *	12253 ± 701 *	1.8	0.8	n/d
2 MPC Zn + 2 MPC Cd	56876 ± 1029 *	10574 ± 496 *	5.4	4.4	575 ± 81 *
5 MPC Zn + 5 MPC Cd	19130 ± 693 *	19130 ± 783 *	1.0	n/d	n/d

±—the standard deviation (SD); * significant at 0.050 probability level.

**Table 3:** Cytomorphometric indices of the *H. vulgare* leaf lamina grown on the soil at different levels of individual and combined Zn and Cd soil contamination.

Rate of Application, mg/kg	Number of Chlorenchyma Cells Per 1 μm^2^ of Cross-Section	Ratio Between Cells’ Cross-Section to Inter-Cell Space	The Average Chlorenchyma Size, µm^2^	Number of Laminas in a Parenchyma Cell
Control	2981 ± 215	0.6	124 ± 16	6.7 ± 0.8
2 MPC Zn	2791 ± 186	0.6	129 ± 12	6.4 ± 0.8
5 MPC Zn	2759 ± 195	0.5	122 ± 11	6.0 ± 0.7
2 MPC Cd	2901 ± 208	0.6	131 ± 15	5.9 ± 0.9
5 MPC Cd	2659 ± 173	0.5	129 ± 14	5.0 ± 0.7 *
2 MPC Zn + 2 MPC Cd	2871 ± 205	0.6	136 ± 15	5.6 ± 0.8
5 MPC Zn + 5 MPC Cd	2810 ± 191	0.5	147 ± 19	4.4 ± 0.9 *

±—the standard deviation (SD); * significant at 0.050 probability level.

## Data Availability

Not applicable.
